# Age trend of the male to female sex ratio in surgical gastric cancer patients at a single institution

**DOI:** 10.1186/1477-7819-12-269

**Published:** 2014-08-21

**Authors:** Junxiu Yu, Yongjun He, Zhen Guo

**Affiliations:** Department of Gastrointestinal Surgery, Liaocheng People’s Hospital and Liaocheng Clinical School of Taishan Medical University, 67 West Dongchang Road, Liaocheng, Shandong Province 252000 China; Department of General Surgery, Jurong People’s Hospital, 60 West Avenue, Huayang, Jurong, Jiangsu Province 212400 China

## Abstract

**Background:**

In previous reports concerning the association between sex disparity and age, gastric cancer (GC) patients were simply divided into younger and older groups by age. We analyzed the age trend of the male to female sex ratio (MFSR) in GC based on patient sequential age in order to observe the changing process of MFSR with age.

**Methods:**

One thousand seven hundred fifty-one surgical gastric adenocarcinoma patients aged 26 to 85 years were investigated between January 1996 and December 2010. The patients were grouped by age intervals of 5 years. The Cochran-Armitage trend test was used to determine how the MFSR changed with age.

**Results:**

The median age of the 1,751 patients with GC was 60 years (26 to 85 years). There were 1,334 male and 417 female patients (MFSR was 3.20). Cochran-Armitage trend test analysis showed that total MFSR increased significantly with age (Z = 5.964, *P* < 0.0001). Further studies on age groups of 26 to 60 years and 61 to 85 years were conducted. The trend test showed that MFSR increased significantly with age from 26 to 60 years (Z = 7.433, *P* < 0.0001). However, MFSR did not increase in ages 61 to 85 years (Z = -0.607, *P* = 0.544).

**Conclusions:**

MFSR in GC presented an increasing trend until 60 years of age. The male GC patients showed an increasing tendency, and female GC patients showed a decreasing tendency with age. This trend reached a plateau phase after 60 years of age.

## Background

Gastric cancer (GC) is among the most common malignant tumors worldwide, even taking into consideration that the incidence of GC has recently decreased in some countries. In 2008, nearly 995,000 new GC patients were identified worldwide. Among these new cases, a total of 646,000 were male and 349,000 were female. The male-female sex ratio (MFSR) was 1.851 [1]. A total of 377,000 new GC cases were estimated to occur in China in 2005; the MFSR was 2.04 [2]. Similar results were reported by GLOBOCAN 2008; the male-female sex ratio was 1.84 and 2.13 in the world and China, respectively. Additionally, the male cases were more numerous compared to female cases in hospitalized GC patients. The MFSR from a large series of data for Japanese surgical patients with GC that was collected from 187 hospitals in 2001 was 2.29:1 [3]. Jeong *et al*. [4] reported that MFSR was 2.05:1 in data from 14,658 surgical GC patients that were collected from 59 Korean institutions in 2009. The MFSR of 1,451 GC patients who underwent surgery from 1999 to 2003 from a single Chinese institute was 2.19:1 [5].

However, male GC patients were not predominant at every age. Qiu *et al*. [6] reported that the MFSR increased with an increase in age. The total MFSR was 2.15:1. But, the MFSR of young GC patients (aged 50 years or younger) was 1.39. The MFSR of older GC cases (over 50 years of age) was 2.64. Chung *et al*. [7] analyzed 3,242 young patients with GC between 18 and 45 years of age. The total MFSR was 1.17. The MFSR were 0.63, 1.01, and 1.67 in 18 to 30-year, 31 to 40-year, 41 to 45-year age groups, respectively. Thus, the MFSR was lower in young GC patients compared to older GC patients.

The above-mentioned studies demonstrated that there are sex disparities in GC patients in different age groups. There may be more female GC patients compared to males at an early age. Therefore, the number of female GC patients decreases with age. However, in previous studies concerning the association between sex disparity and age, the patients were simply divided into younger and older groups by age. It has remained unclear how the MFSR changed; moreover, the time point at which the change stops has not been elucidated. Thus, in this study, we analyzed the changing process of MFSR by sequential GC patient age in order to observe the age trend of MFSR in GC. And, we hope our study could potentially be helpful to explore the factors that influence the pathogenesis of GC in female adults.

## Methods

### Patient population

Data were obtained from surgical patients from Liaocheng People’s Hospital (Shandong, China) from January 1996 to December 2010. All medical records of cases diagnosed with GC were reviewed. The patients with gastric adenocarcinoma were selected. The patients with GC of other histological types were eliminated. A total of 1,758 cases with medical records that satisfied the study requirements were investigated. All patients were grouped by interval ages of 5 years. Five patients (three cases were male, two were female) aged 20 to 25 years, and two patients (one from each sex) aged 86 to 88 years, were excluded from the study because the number of patients was too small to complete an age group. Finally, a total of 1,751 patients with gastric adenocarcinoma were included in this study. The study was approved by the Ethical Committee of Liaocheng People’s Hospital.

### Statistical analysis

The Cochran-Armitage trend test was used to identify the age trend of MFSR. All analyses were performed using SAS 9.0 software (SAS Institute Inc., Cary, NC, USA). Statistical significance was defined as a *P* value less than 0.05. All statistical tests were two-sided.

## Results

**Figure 1:**
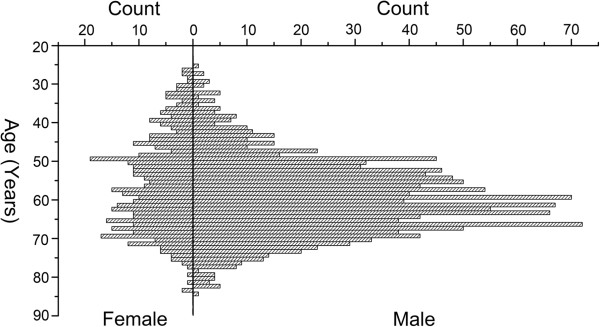
**A histogram of male and female gastric cancer patients by age.**

The median age of 1,751 patients with gastric adenocarcinoma was 60 years (26 to 85 years). A histogram of all patient ages according to sex is shown in Figure 1; there were 1,334 male and 417 female patients. The MFSR of all GC patients was 3.20. The median age of patients was 58 years (27 to 84 years) in females and 61 years (26 to 85 years) in males.

**Figure 2:**
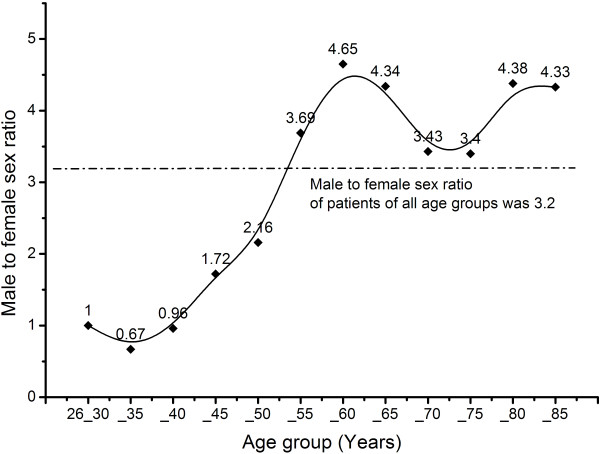
**The male-female sex ratio (MFSR) of each age group patients with gastric cancer, and the Basis spline.**

The Cochran-Armitage trend test showed that the MFSR increased significantly with age (*Z* = 5.964, *P* < 0.0001) in total GC cases. The MFSR curve (Basis spline) by each age group is shown in Figure 2.

The Cochran-Armitage trend test was used to analyze whether there was a variation in trend for MFSR from ages 26 to 60 years and from ages 61 to 85 years. The results showed that MFSR increased significantly with age from 26 to 60 years (*Z* = 7.433, *P* < 0.0001). However, MFSR did not increase with age from 61 to 85 years (*Z* = -0.607, *P* = 0.544). As shown in Figure 2, the MFSR increased until the 56 to 60 age group, and MFSR reached a plateau after this age group.

## Discussion

To the best of our knowledge, this study is the first to analyze the age trend of MFSR in GC using a trend test based on patient sequential age. These results, which are based on continuous data, are more reliable compared to previous studies that divided patients simply into younger and older age groups. The results show that MFSR increased gradually with age. The percentage of female GC cases gradually decreased with age. The percentage of male GC cases gradually increased with age.

Compared to male GC patients, female patients exhibited a decreasing trend with age. Our results are in concordance with data from the National Cancer Center of China. The data from 2003 to 2007 showed that the male to female incidence ratio of GC was elevated with age [8]. Rutegard *et al*. [9] found similar results based on a study of the sex disparities in GC incidence in Sweden from 1970 to 2006. The sex (male to female) ratios of gastric non-cardia gastric adenocarcinoma presented increasing tendency in the 1970 to 1986 and 1987 to 2006 periods.

The MFSR did not continue to increase after 60 years; it reached a plateau in our study. The Cochran-Armitage trend test showed that the MFSR increased gradually in the 26 to 60 years group but did not increase in the 61 to 85 years group. Our results are supported in a study by Rutegard *et al*. [9]. They reported the sex (male to female) ratios of non-cardia GC patients increased steadily with age and reached a plateau in the age group 65 to 74 years. The male to female ratio of incidence of GC in China present as an ‘S" type [8]. The ratio remained almost the same before 30 years of age and began to increase markedly at 30 years of age, reaching a plateau after 60 years. Thus, the MFSR in our study was in conformity with the incidence of GC in China. It is unfortunate that the trend of MFSR before 30 years of age could not statistically analyzed in our study due to limited data.

There are few studies of the trend of MFSR in GC. This study did not evaluate why the proportion of female GC patients showed a decreasing tendency and the proportion of male GC patients showed an increasing tendency with age. Presumably, male individuals experience a greater attack from environmental pathogenic factors (such as *H. pylori* infection, high salt diet, smoking, *etcetera*) compared to females by the time adulthood is reached. Some studies [10] have suggested that a later acquisition of *H. pylori* gastritis in females compared to males causes later onset of GC. Therefore, the abovementioned fact may explain why the proportion of male GC patients tended to increase after 30 years of age.

Another reason may be the protective effect of estrogen for GC [11–14]. Duell *et al*. [15] found that women with an ovariectomy had a 79% increased risk of GC compared with women who had no ovariectomy. A population-based prospective study of women aged 40 to 70 years was conducted based on 73,442 Shanghai women with 154 GC patients [16]. The results showed that there were no associations between GC risk and age of menarche, number of children, breast feeding, or oral contraceptive use. However, there were associations between GC risk and age of menopause and years of fertility. Recently, a meta-analysis demonstrated that exposure to ovarian or exogenous estrogen may decrease the risk of GC [17]. Longer years of fertility (RR = 95% CI: 0.63 to 0.86) and hormone replacement therapy decreased GC risk (RR = 0.74, 0.77, respectively). However, the age at menarche, age at menopause, years of fertility, parity, age at first birth, and oral contraceptive use were not found to be associated with GC risk. A Korean multicenter cancer cohort study demonstrated that isoflavone and phytoestrogens were associated with decreased risk for GC [18]. Some reports have indicated that treatment with tamoxifen, an antiestrogen, increased the incidence of GC in women and men [12, 19].

## Conclusions

In conclusion, Cochran-Armitage trend test analysis indicated that the proportion of female GC patients showed a decreasing tendency and proportion of male GC patients showed an increasing tendency with age. However this trend stops after 60 years of age.

## Abbreviations

GC: gastric cancer
MFSR: male to female sex ratio.
